# Emerging roles and the regulation of aerobic glycolysis in hepatocellular carcinoma

**DOI:** 10.1186/s13046-020-01629-4

**Published:** 2020-07-06

**Authors:** Jiao Feng, Jingjing Li, Liwei Wu, Qiang Yu, Jie Ji, Jianye Wu, Weiqi Dai, Chuanyong Guo

**Affiliations:** 1 Department of Gastroenterology, Putuo People’s Hospital, Tongji University School of Medicine, number 1291, Jiangning road, Putuo, Shanghai, 200060 China; 2 Department of Gastroenterology, Shanghai Tenth People’s Hospital, Tongji University School of Medicine, number 301, Middle Yanchang road, Jing’an, Shanghai, 200072 China; 3 Department of Gastroenterology, Zhongshan Hospital of Fudan University, Shanghai, 200032 China; 4 Shanghai Institute of Liver Diseases, Zhongshan Hospital of Fudan University, Shanghai, 200032 China; 5 Shanghai Tongren Hospital, Shanghai Jiaotong University School of Medicine, Shanghai, 200336 China

**Keywords:** Hepatocellular carcinoma, Aerobic glycolysis, HK2, PFK1, PKM2, HIF-1α

## Abstract

Liver cancer has become the sixth most diagnosed cancer and the fourth leading cause of cancer death worldwide. Hepatocellular carcinoma (HCC) is responsible for up to 75–85% of primary liver cancers, and sorafenib is the first targeted drug for advanced HCC treatment. However, sorafenib resistance is common because of the resultant enhancement of aerobic glycolysis and other molecular mechanisms. Aerobic glycolysis was firstly found in HCC, acts as a hallmark of liver cancer and is responsible for the regulation of proliferation, immune evasion, invasion, metastasis, angiogenesis, and drug resistance in HCC. The three rate-limiting enzymes in the glycolytic pathway, including hexokinase 2 (HK2), phosphofructokinase 1 (PFK1), and pyruvate kinases type M2 (PKM2) play an important role in the regulation of aerobic glycolysis in HCC and can be regulated by many mechanisms, such as the AMPK, PI3K/Akt pathway, HIF-1α, c-Myc and noncoding RNAs. Because of the importance of aerobic glycolysis in the progression of HCC, targeting key factors in its pathway such as the inhibition of HK2, PFK or PKM2, represent potential new therapeutic approaches for the treatment of HCC.

## Background

According to the latest global cancer statistics (2018), liver cancer has become the sixth most commonly diagnosed cancer and the fourth leading cause of cancer death worldwide in 2018 [1]. Because of the high infection rates of hepatitis B virus (HBV) and hepatitis C virus (HCV), the morbidity and mortality due to liver cancer is increasing in China [2]. Primary liver cancer is composed of hepatocellular carcinoma (HCC) (75–85%) and intrahepatic cholangiocarcinoma (10–15%), as well as other rare types [3]. The risk factors associated with primary HCC includes chronic HBV/HCV infection, aflatoxin intake, alcohol abuse, smoking, obesity, and others [4]. Since the early-stage HCC is often asymptomatic, patients with HCC are usually diagnosed at intermediate or advanced stages, and miss the opportunity for curative treatment (hepatic resection or liver transplantation) [5].

For patients with advanced HCC, treatment options include local ablation, radiotherapy, chemotherapy and molecular targeted therapies [6]. Among them, sorafenib, a multi-target tyrosine kinase inhibitor, is the first line drug approved for the treatment of advanced HCC by the Food and Drug Administration (FDA) in 2005 [7, 8]. The famous Sorafenib Hepatocellular Carcinoma Assessment Randomized Protocol (SHARP) trial demonstrated that sorafenib improved patient overall survival for an extra 2.8 months [9]. However, the efficacy of sorafenib is limited owing to the development of drug resistance within 6 months [10]. The mechanisms responsible for sorafenib resistance are complex, but includes the activation of epidermal growth factor receptor (EGFR), c-Jun and the Akt pathway, as well as increases in cancer stem cells, and enhancement of epithelial-mesenchymal transition (EMT) [11].

There are six hallmarks of cancer, including sustained proliferative signaling, evasion of growth suppression, resistance to cell death, replicative immortality, inducing angiogenesis, and activation of invasion and metastasis [12], all of which contribute to the malignant biological properties of cancer. Recently, new theories are emerging that energy metabolism reprogramming may be another hallmark of cancer [13]. In the 1920s, Otto Warburg firstly showed that unlike normal cells that catabolize glucose by oxidative phosphorylation in the mitochondria, tumor cells tend to convert glucose into lactate even in conditions of sufficient oxygen [14]. This phenomenon was termed as aerobic glycolysis or the Warburg effect, and is characterized by enhanced glucose uptake and lactate production. Although the adenosine triphosphate (ATP) production efficiency is low during aerobic glycolysis, it still takes up to 50–70% of the ATP supply in different tumors [15]. Furthermore, the metabolic intermediates generated during aerobic glycolysis can be used for the biosynthesis of biomacromolecules used by the tumor to meet the demands for rapid growth [16]. The production of lactate also provides an acidic environment to aid the invasion and metastasis of cancer [17]. Moreover, some researchers have found that increases in aerobic glycolysis are also involved in sorafenib resistance [18].

Aerobic glycolysis plays an important role in the proliferation, growth, invasion and treatment of cancer. A better understanding of aerobic glycolysis in HCC will help to reveal the pathogenesis and potential treatment paths for HCC, as well as the mechanism of sorafenib resistance [19]. Therefore, this review aimed to review the characteristics and regulatory mechanisms of aerobic glycolysis in HCC, and to identify potential new therapeutic targets for its treatment.

## Characteristics of aerobic glycolysis in HCC

### Enhancement of aerobic glycolysis in HCC

The Warburg effect was firstly reported in rat liver carcinoma in the 1920s. Warburg and his co-workers found that rat liver carcinoma did not consume more oxygen(O_2_) than normal liver tissue; but instead, even in the presence of sufficient O_2_, liver carcinoma tissue also converted glucose and pyruvate into lactate, rather than transferred pyruvate into the mitochondria for use in the citric acid cycle. Moreover, Cori C. F. and Cori G. T. further reported that the blood drawn from a Rous sarcoma tumor containing veins showed significantly less glucose and more lactate than normal tissues [14]. After these initial findings, this enhancement of aerobic glycolysis has been found in many other cancer types, including breast cancer [20], renal cell carcinoma [21], pancreatic cancer [22], lung cancer [23], gastric cancer [24], and prostate cancer [25]. The existence of aerobic glycolysis in HCC has also been proved by Li S [26], Beyoğlu D [27] and Bustamante E [28], which found that the aerobic glycolysis was enhanced in HCC as well as hepatoma.

Warburg originally hypothesized that the mitochondrial respiration (also known as oxidative phosphorylation, OXPHOS) must be damaged in cancer cells because they used high levels of O2, and found that they were unable to suppress lactate production in cancer cells. However, Chance and Weinhouse in 1950s firstly found that there were no mitochondrial defects in cancers, opposing to Warburg’s conception [14]. Currently, many researches still believe that the mitochondria are not injured in cancers and OXPHOS remains functional for the supply of O_2_ [29]. However, there have also been studies reporting mutations in mitochondrial DNA (mtDNA), specifically those genes coding for proteins involved in OXPHOS, such as point mutation (52% of HCC patients), gene deletions or insertions, and copy number changes [30, 31], and there exists a dysfunction in coenzyme Q10 [32] and other components of the mitochondrial electron transport chain [33]. Drugs targeting mitochondria, such as oligomycin, combined with anti-glycolysis drugs, such as 2-deoxyglucose (2-DG), showed a synergic effect in triggering cancer cells death [34].

Although aerobic glycolysis has been observed in many cancers, most cancer cells do not utilize aerobic glycolysis alone. Instead, they consume ATP from both the mitochondria OXPHOS and the aerobic glycolysis. In most normal cells, the ATP produced from mitochondria OXPHOS and glycolysis is approximately 90 and 10%, respectively. Whereas cancer cells rely on aerobic glycolysis to provide as much as 60% of the ATP consumption [35]. It’s well known that the mitochondrial OXPHOS can produce 36 molecules of ATP, while the use of glycolysis to synthesis lactate can just produce only 2 molecules of ATP. Although the aerobic glycolysis seems to be energetically inefficient in the production of ATP, this is compensated for by the fact that the aerobic glycolysis process is more rapid and it also generates further downstream biomacromolecules required for cell proliferation [36]. Rapid glucose fermentation by glycolysis also causes cancer cells to take up more glucose than normal cells [37]. These findings suggest that the use of aerobic glycolysis in HCC provides advantages during cancer progression.

### Key enzymes in aerobic glycolysis in HCC

**Fig. 1:**
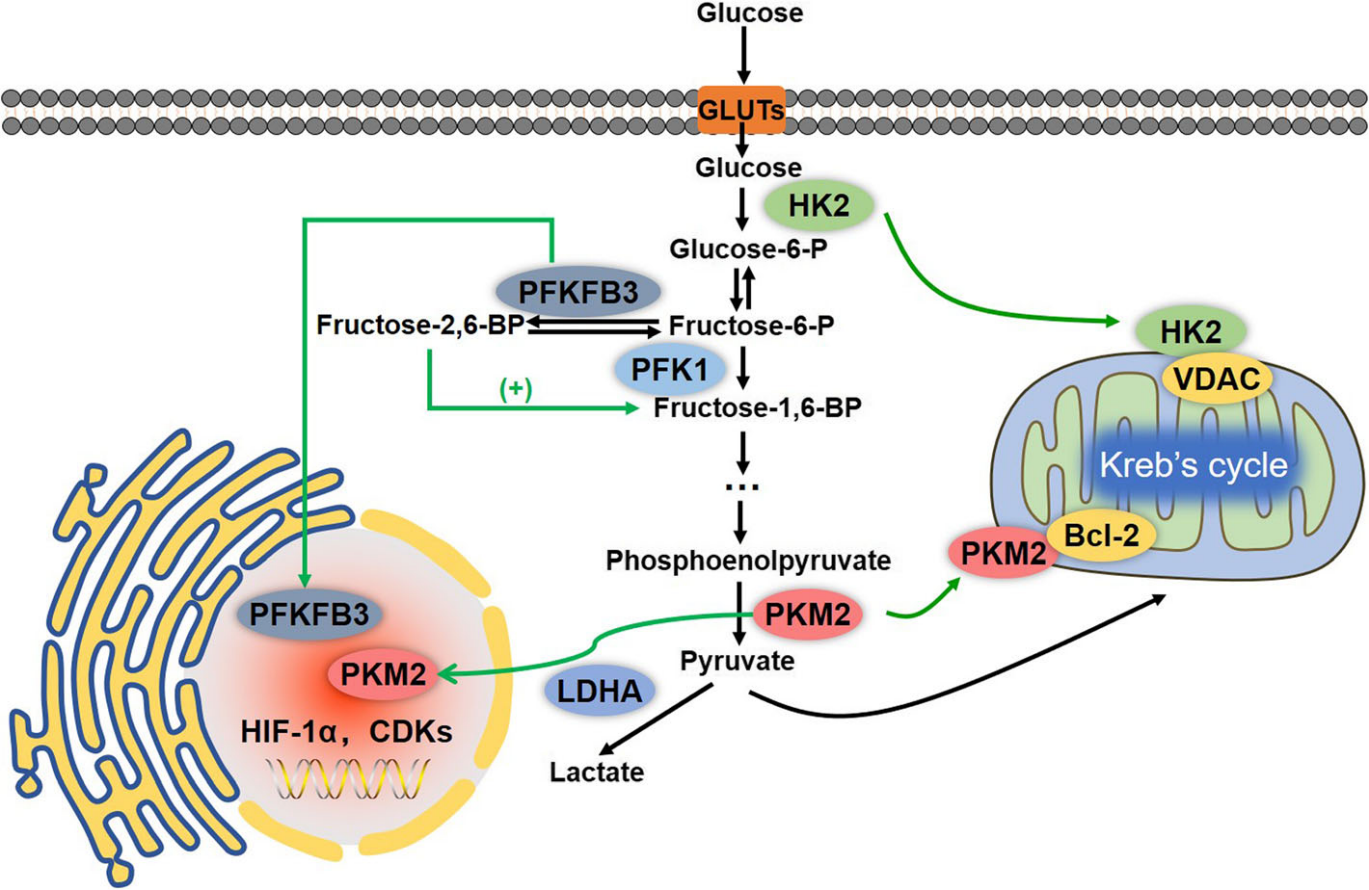
Aerobic glycolysis process and its three rate-limiting enzymes. Aerobic glycolysis was originally found in HCC and is the process that converts glucose into pyruvate and lactate instead of using OXPHOS even in sufficient O_2_ supply. HK2, PFK1 and PKM2 are the three rate-limiting enzymes involved in glycolysis. HK2 catalyzes glucose to G-6-P, and can interact and bind with VDAC1 in the mitochondrial outer membrane to facilitate the production of ATP and the inhibition of apoptosis. PFK1 can catalyze F-6-P to F-1,6-BP, and its activity can be regulated by PFKFB3 catalyzed products F-2,6-BP. PKM2 not only catalyzes PEP to pyruvate, but can also translocate into the nucleus and act as a co-activator for some transcription factors, such as HIF-1α, β-catenin/c-Myc, NF-κB and STAT3, to promote the transcription of relevant target genes

There are three rate-limiting enzymes in aerobic glycolysis, including hexokinase (HK), phosphofructokinase (PFK), and pyruvate kinases (PKs) (Fig. 1). The expression changes of these enzymes can largely influence the progression of HCC.

#### Hexokinase 2 (HK2)

Hexokinase (HK) is the first rate-limiting enzyme in aerobic glycolysis and can catalyze the conversion of glucose to glucose-6-phosphate (G-6-P) [38]. The enzymes have four isoforms (HK1, HK2, HK3 and HK4), but most normal tissues express only HK1. However, HK2 is highly expressed in HCC tissues and it is directly linked to pathological stage and patient prognosis [39].

HK2 is more efficient at promoting aerobic glycolysis than the other isoforms [40], and the mechanisms by which HK2 promotes glycolysis has been investigated by many researchers. Firstly, HK2 interacts and binds to the voltage-dependent anion-selective channel protein 1 (VDAC1) in the mitochondrial outer membrane, then facilitates the activation of ATP synthesis-related enzymes to enhance the production of ATP and the inhibition of apoptosis (Fig. 1) [41]. Secondly, when HK2 is bound to VDAC1, it is protected from the inhibitory effects of its downstream products, such as G-6-P, thereby enhancing the glycolysis process and the rate of ATP production [15]. The expression of HK2 is controlled by several signaling pathways and transcription factors, including the PI3K/Akt/HIF-1α axis, β-catenin/c-Myc signaling pathway, STAT3, and miR-199a [42–45].

Recently, a novel isoform has been found termed hexokinase domain containing 1 (HKDC1). Normally, HKDC1 is upregulated in pregnant women at 24–28 weeks of gestation and regulates the whole-body glucose homeostasis during pregnancy through its role in glucose use [46]. However, Zhang et al. reported in 2016 that HKDC1 is also overexpressed in HCC and is associated with a lower overall survival, possibly through the upregulation of the Wnt/β-catenin pathway, causing increased HCC cell proliferation and migration [47].

It has been reported that genetic liver-specific knockout of HK2 decreases the proliferation and the formation of HCC induced by diethylnitrosamine. HK2 depletion inhibits glycolysis flux and induced OXPHOS, enhancing the sensitivity of HCC to drugs, such as metformin. Moreover, HK2 silencing can synergistically enhance the sensitivity of HCC to sorafenib, thereby inhibiting tumor growth in mice [39]. Based on the key role of HKs in HCC, HK2 is considered to be a highly promising metabolic target for the development of new treatments for HCC.

#### Phosphofructokinase-1 (PFK1)

Phosphofructokinase-1 (PFK1) is the second rate-limiting enzymes involved in glycolysis, and can catalyze fructose 6-phosphate(F-6-P) to fructose 1,6-bisphosphate (F-1,6-BP) using ATP [48]. There are three isoforms of PFK1 in mammals, which are PFK-M (found in muscle), PFK-P (found in plasma) and PFK-L (found in liver), and the proportion of these isoforms may vary in different tissues depending on their specific energy metabolism requirements [49]. In liver, it has been reported that expression levels of PFK-L, PFK-M, and PFK-P subunits are expressed in this descending order in humans and in rats [48].

PFK1 is primarily synthesized as an unstable and inactive monomer, which can then rapidly form dimers, which maintains the minimal catalytic activity for F-6-P [50]. However, the fully activated PFK1 is present as a tetramer, and the formation and stabilization of PFK1 tetramers largely influences the glycolytic flux rate largely [51]. However, the downstream products of PFK1 enzymes, including ATP, phosphoenolpyruvate (PEP), citric acid and lactate, can induce the dissociation of PFK1 tetramers to dimers, leading to the inhibition of PFK enzymatic activity and providing a negative feedback to the glycolytic process [52]. However, increased PFK-1 activity has been found to promote glycolysis and proliferation in cancer cells [53]. For example, fructose - 2,6 - bisphosphate (F-2,6-BP), which is also the product of F6P catalyzed by PFKFB3 (Fig. 1), is considered to be the most potent allosteric activator of PFK1, and can increase PFK1 activity even in the presence of ATP [51].

As mentioned above, the enzymes 6-phosphofructo-2-kinase/fructose-2,6-bisphosphatase-3 (PFK2/PFKFB-3) play a significant role in the regulation of glycolysis in HCC, as well as in tumor growth and metastasis [52, 54]. PFKFB3 can translocate into the nucleus to regulate the activity of cyclin-dependent kinase (CDKs), resulting in the arrest of the cell cycle and the inhibition of cell death (Fig. 1) [55]. Moreover, overexpression of PFKFB3 can increase the expression of VEGF-A, thereby promoting angiogenesis and facilitating metastasis in breast cancer [56]. By inhibiting PFKPB3, aspirin in combination with sorafenib, overcomes sorafenib resistance by inducing apoptosis in HCC [54]. The expression of PFKFB3 can also be regulated by hypoxia inducible factor-1α (HIF-1α) mediated transcription, AKT, and PTEN [57].

In many cancer types, high levels of PFKFB3 are correlated with poor prognosis with lymph node metastasis or poor survival [58, 59]. Therefore, targeting PFKFB3 can also be a new therapeutic approach for the treatment of HCC.

#### Pyruvate kinases, type M2 (PKM2)

The last rate-limiting enzymes in glycolysis process are pyruvate kinases (PKs), which catalyze PEP to produce ATP and pyruvate. There are also four isoforms of PKs, including liver-type PK (PKL), red blood cell PK (PKR), and PK muscle isozymes M1 and M2 (PKM1 and PKM2, respectively) [60]. Unlike other forms of PKs, PKM2 is highly up-regulated in cancer cells, and is associated with a poor prognosis [35].

**Fig. 2:**
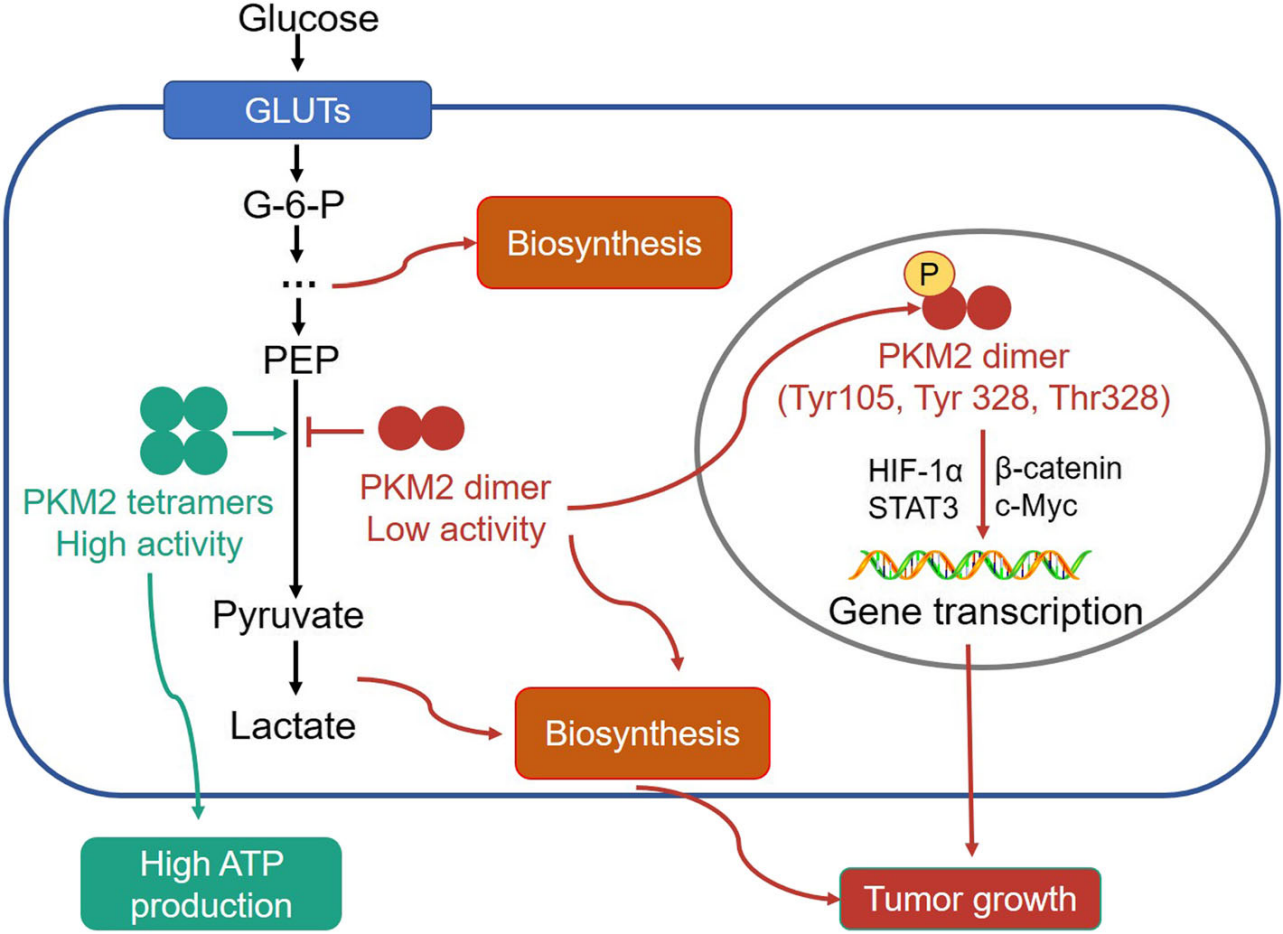
The activity of PKM2 is dependent upon its oligomerization states. The PKM2 tetramer exhibits high levels of pyruvate kinase activity and can accelerate the transformation of PEP to pyruvate, thereby increasing the glycolytic flux and ATP production rates. Whereas PKM2 in dimeric form exhibits lower levels of pyruvate kinase activity, and can be phosphorylated at Tyr105, Tyr 328, Thr328 or Pro403/408 sites and then translocate into nucleus to act as a co-activator for some transcription factors, such as HIF-1α, β-catenin/c-Myc, NF-κB and STAT3, leading to tumor progression

The activity of PKM2 is mainly controlled by its oligomerization states (Fig. 2). There are two forms of PKM2, one is a tetramer, with higher catalytic activity, and is located in the cytoplasm and can transform PEP to pyruvate rapidly, the glycolytic flux and the production of more ATP [61]. However, the other isoform is a monomer or dimer, with lower catalytic activity, and can translocate into the nucleus to act as a co-activator of several transcription factors, such as HIF-1α, β-catenin/c-Myc, NF-κB and STAT3 [60, 62]. Once in the nucleus, PKM2 can promote the transcription of target genes, such as HIF-1α targeted expression of GLUTs, PKM2, LDH-A, and VEGF-A, leading to the promotion of growth, positive feedback regulated-glycolysis and angiogenesis in cancer cells [63].

The activity of PKM2 can also be regulated by numerous allosteric effectors and post-translational modifications. For example, the upstream metabolic intermediates F-1,6-BP can act as an allosteric activator to increase the activity of PKM2; while the downstream products such as ATP and L-cysteine can inhibit PKM2 activity [64–66]. PKM2 can also be post-translationally modified. The phosphorylation at Tyr105, Tyr 328, Thr328 or Pro403/408 sites, or the succinylation at K498 site, or acetylation at K433 site of PKM2 has been demonstrated to inhibit the tetramer formation and activity of PKM2, but promotes its nuclear translocation [64, 65, 67, 68]. Moreover, PKM2 can also interact and bind with some oncogenic proteins, including pp60v-src, to increase dimer formation [69]. Heterogeneous ribonucleoprotein (hnRNP) also influences the alternative splicing of PKM genes, giving rise to differences in the PKM1/PKM2 ratio [70]. Some signaling pathways, such as HIF-1α, PI3K/mTOR and PPAR-γ, also up-regulate the expression of PKM2 to promote the growth of cancer cells [71].

Since its discovery in HCC, and the over-expression of PKM2 has been found in many other cancer types, including cervical cancer, lung cancer, breast cancer, colorectal cancer, and prostate cancer, especially the lower activity dimer form of PKM2 [72]. Recently, it has been reported that the expression levels of PKM2 are associated with the clinicopathological features of HCC, such as the size, the number and the clinical stages of tumors. HCC patients with higher levels of PKM2 expression exhibit a higher cumulative recurrence rate than those with lower PKM2 levels [73]. By switching from PKL to PKM2, HCC cells can elevate glucose uptake levels and increase the oxidative stress [74]. There are also some specific inhibitors, such as compound 3 k and shikonin, which have the ability to inhibit the formation of PKM2 tetramers, thereby inhibiting the growth of HCC, and making PKM2 another therapeutic target for the treatment of HCC [75, 76].

### Role of enhanced aerobic glycolysis in HCC

#### Proliferation, growth and immune evasion in HCC

As a new hallmark of HCC, aerobic glycolysis is believed to promote the proliferation, growth, and induce immune evasion in HCC through the following evidence: (1) there is rapid ATP production during aerobic glycolysis, enabling the tumor to adapt to its microenvironment which is short of energy resources [41]. (2) The enhanced aerobic metabolism is accompanied by activated glycolytic flux, with increased amounts of metabolic intermediates production, such as dihydroxyacetone phosphate (DHAP, which can be used for the synthesis of triglycerides and phospholipids), 3-phosphoglycerate (3-PG, which can be used for the synthesis of amino acids). These can then enter other metabolic pathways for the synthesis of nucleotides, lipids, and proteins, enabling cancer cell proliferation [77]. Cancer cells also elevated the aerobic glycolysis to promote glutaminolysis to satisfy the precursor needs required for nucleic acids biosynthesis [78]. (3) The production of lactate, as well as the hydrogen ions (H^+^) produced during these processes, cause the acidification of the extracellular environment, which inhibits the function of immunosuppressive cells, including M2 type macrophages and lymphocytes, further facilitating the survival of cancer cells [36].

Many studies have found that the pro-survival effect of aerobic glycolysis in HCC is caused by the inhibition of enzymes involved in glycolysis. For example, in xenograft models, HK2 knockdown by shRNA can reduce the growth rates of Hep3B liver tumors by approximately 50% [26]. Furthermore, inhibition of PFKFB3 using specific inhibitors or shRNA, suppresses the growth of HCC both in vivo and in vitro [54]. Knockdown of PKM2 by siRNA also inhibits the proliferation of HCC cell lines [79], and the downregulation of LDH-A can also induce apoptosis and growth arrest in a HCC xenograft mouse model [80].

#### Invasion and metastasis in HCC

The malignant and aggressive nature of cancers can be reflected by the extent of metastasis and invasion. Many studies have found that the metastasis and invasion of HCC are correlated with the enhanced aerobic glycolysis in HCC. For example, Li et al. found that in different HCC cell lines, those that exhibited the greatest invasion capability, including MHCC-97H, HCC-LM3, showed a high level of aerobic glycolysis when compared to less invasive cell lines such as HepG2 [54, 75, 81]. These phenomenon were also common in breast cancer, prostate cancer, cervical and head and neck cancers [82].

Because of the hypoxic nature of tumor tissues, cancer cells tend to metastasize to additional sites to enhance energy and blood supplies, thereby enabling its survival. The glycolytic phenotype aids this metastasis and invasion in HCC mainly through lactate and H^+^ mediated acidification of the extracellular environment, which includes the following aspects: (1) the low pH in extracellular environment causes destruction of the normal tissue through caspase-mediated or p53-dependent apoptosis [83]. (2) Acidification of the extracellular matrix (ECM) promotes the secretion of proteolytic enzymes such as cathepsin B or metalloproteinases, so as to help the degradation of ECM and facilitate metastasis [82]. (3) The immunosuppression caused by low pH levels also enables metastatic cancer cells to escape the surveillance of immune system, leading to the sustained metastasis [84]. As a result, aerobic glycolysis is responsible for the extracellular structure and immunosuppression, making it easy for cancer cells to metastasize and invade.

#### Angiogenesis in HCC

Angiogenesis, also known as neovascularization, is a pathologically abnormal vessels formation in HCC, which supply the necessary requirement for rapid tumor growth and initiation of metastasis [85]. Angiogenesis in HCC is also driven by hypoxia. As mentioned above, HCC is a solid tumor, and its structure consists of a tumor surrounding stroma, basal membrane, tumor tissues, and central necrosis [82]. Therefore, there is hypoxia in tumor tissues due to its specific tumor tissues structures. However, though the angiogenic factors, such as vascular endothelial growth factor (VEGF) and the angiopoietin families (Angs), can be released by hypoxia in cancer cells, the new vessels remain physically separated from cancer cells due to the existence of basement membranes. Therefore, although HCC is largely dependent on angiogenesis for its energy supply during invasion and metastasis, the metastasis and invasion processes also favor the angiogenesis by destroying the ECM structures [86].

In fact, it’s believed that the ‘glycolytic switch’ occurs before the angiogenesis [82]. Aerobic glycolysis also causes angiogenesis process in HCC through many pathways. For example, the acidification of the extracellular environments by lactate and H^+^ can promote the secretion of VEGF and interleukin 8, which are both angiogenic factors and can induce angiogenesis [87, 88]. Moreover, the production of pyruvate by glycolysis can also induce the expression of HIF-1α and accelerate the angiogenesis process by mediating the transcription of VEGF and plasminogen activator inhibitor-1 (PAI-1) in HepG2 cells. HIF-1α can also promote the expression of the glycolytic enzymes, such as PKM2, HK2 and LDHA to promote glycolysis in tumors, which forms a positive feedback loop for tumor progression [88].

#### Drug resistance in HCC

The enhancement of aerobic glycolysis can also contribute to drug resistance in HCC. It has been reported that high glycolysis levels were significantly associated with poor prognosis in cancer chemotherapy in combination with bevacizumab [89]. Furthermore, Li et al. and Feng et al. found that the aerobic glycolysis was enhanced in different HCC cell lines, and was associated with sorafenib resistance. By downregulating PFKFB3 or PKM2, the sorafenib resistance seen in HCC can also be improved [54, 75, 90]. Moreover, combined treatment with sorafenib and 2-DG, which is a HK2 inhibitor, can synergistically suppress the proliferation of sorafenib resistant HCC cells by inhibiting ATP production [91].

The mechanisms by which aerobic glycolysis influences drug sensitivity in HCC can be concluded as: (1) HK2 can enter the mitochondria and interact with VDAC, inhibiting the release of cytochrome c and subsequent apoptosis, leading to the inhibition of cell death [92]. (2) Some signaling pathways, such as the PI3K/Akt/mTOR pathway, can activate HK2 and PKM2 in cancer cells and promote their survival and drug resistance [93]. (3) The pyruvate dehydrogenase (PDH), which catalyze pyruvate into acetyl coenzyme A, can be inactivated by PDH kinase and then promote the synthesis of lactate. The decreased level of PDH has been reported to be responsible for sorafenib-acquired resistance in HCC, and this effect can be reversed by using a PDH kinase inhibitor [94]. (4) PKM1 is also reported to promote aerobic glycolysis through autophagy and cause cancer chemoresistance [95]. (5) HIF-1α and c-Myc also participate in chemoresistance in HCC, as there is elevated expression of HIF-1α and c-Myc in HCC tissues, and these have been found to target the multi-drug resistance (MDR) gene MDR1 [96]. (6) Besides, the immunosuppression caused by acidification of the extracellular environment also contributes to drug resistance [97].

## Regulatory mechanisms of aerobic glycolysis in HCC

### AMP-activated protein kinase (AMPK)

The AMPK is a highly conserved Ser/Thr kinase consisting of catalytic α, regulatory β, and γ subunits, and acts as a key energy status sensor and energy homeostasis regulator, including glucose, protein and lipid metabolism and autophagy [52]. During energetic stress, AMPK inhibits the ATP consuming processes, such as lipid and protein biosynthesis and cell proliferation, while promoting the ATP conserving process, such as autophagy and glycolysis [98]. AMPK is usually activated by the serine/threonine kinase liver-kinase-B1 (LKB1) with Thr172 phosphorylation during energy stress [99]. It has been found that the phosphorylation of AMPK by non-canonical upstream kinases can also affect AMPK activity. For example, the phosphorylation of Ser485 in AMPKα1 and Ser491 in AMPKα2 by PKA, and phosphorylation of Ser491 in AMPKα2 by p70S6 kinase (S6K) can suppress AMPK activity [100, 101].

AMPK plays important roles in the growth, proliferation, autophagy, angiogenesis, metastasis and invasion, and stress response in HCC. For example, Fang et al. reported that the activation of AMPK/mTOR pathway can suppress the HCC malignant phenotype by inhibiting glycolysis [102]; whereas loss of AMPK activation was associated a poor prognosis in HCC patients [103]. Faubert B et al. also reported that genetic ablation of AMPKα1 can accelerate Myc-induced lymphomagenesis and elevate the aerobic glycolysis level, which was through the stabilization of HIF-1α [104, 105]. On the contrary, emerging evidence suggested that AMPK can protect cancer cells from metabolic stress to promote tumor progression [106]. Furthermore, it has been found that the activation of AMPK/mTOR pathway in HCC cells was associated with bile acid induced invasion and migration of HCC [107].

**Fig. 3:**
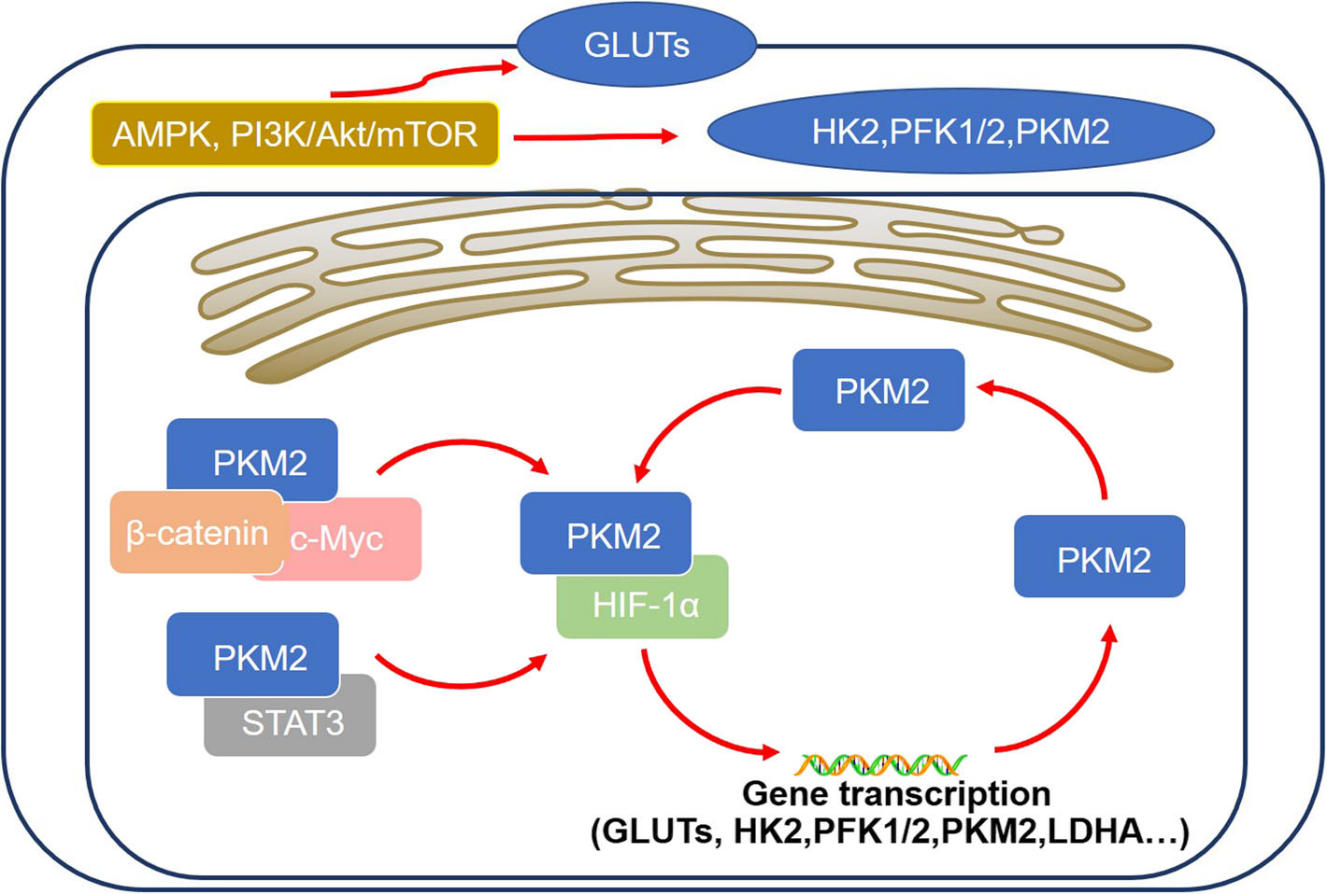
Regulatory mechanisms involved in aerobic glycolysis in HCC. Aerobic glycolysis can be regulated by various transcriptional factors, such as HIF-1α and c-Myc, and many signaling pathways, such as AMPK and PI3K/Akt, as well as noncoding RNAs. The regulatory mechanisms include the regulation of enzymes activity and the relative gene expression levels, and both mechanisms are tightly intertwined

AMPK is a crucial link between metabolism and signaling pathways. The mechanisms by which the activation of AMPK during energetic stress can regulate glycolysis can be concluded as follows. Firstly, AMPK can promote glucose uptake by enhancing the expression of GLUT4 and GLUT1 in cell membrane through the PI3K pathway (Fig. 3) [52, 108, 109]. Secondly, under hypoxia status, AMPK can mediate the activation of PFK2 (PFKFB3) to enhance glycolysis in myocardia. By AMPK-dependent phosphorylation of PFKFB3, the metabolic pattern in tumor cells is switched from oxidative respiration to glycolysis [110]. Thirdly, AMPK has been reported to increase the expression levels of PFKFB3 [108].

AMPK also plays a vital role in autophagy to affect the malignance of HCC. Conventionally, autophagy is activated by mitochondrial depolarization, nutrient starvation, toxic proteins aggregation and infection, leading to deregulation of biomacromolecules for energy supply [111]. However, autophagy is constitutively activated in HCC, and participates in tumorigenesis, metastasis, glycolysis, targeted therapy and drug resistance of HCC through AMPK dependent or independent pathways [112]. Recently, more and more studies have shown that autophagy plays a dual role in HCC. In tumor cells, autophagy protects the survival of tumor cells via the following mechanisms: (1) promotion of metabolite turnover and energy production; (2) inhibition of apoptosis and reactive oxygen species production; and (3) inducing drug resistance [113]. Except for the tumor promotion effect of autophagy, it has also been found that autophagy also has a tumor suppressive effect during the progress of liver cancer. These can be reflected by Qu et al. that knockout of autophagy gene BECLIN1 could induce HCC tumorigenesis in mice [114].

Typically, after the activation of AMPK, mTOR is further inhibited, and then results in the inhibition of autophagy process. Kim et al. reported that under glucose starvation, AMPK promoted autophagy by directly activating ULK1 (an ATG1 homologue) through phosphorylation of Ser 317 and Ser 777. And under nutrient sufficiency, high mTOR activity prevents ULK1 activation by phosphorylating ULK1 Ser 757 and disrupting the interaction between ULK1 and AMPK [115]. Besides, phosphorylation of AMPK suppresses mTORC1 mediated inactivation of Raptor or activation of TSC2 [111]. On the other hand, autophagy can be activated through various AMPK independent pathway, such as the PI3K/AKT/mTOR pathway, MAPK/ERK signaling pathway, transcription factors including c-Myc and p53 [116, 117].

Collectively, these findings provide a further prospect that AMPK is a promising target in the treatment for HCC.

### PI3K/Akt pathway

The phosphoinositide 3-kinases (PI3Ks) signaling pathway play important roles in both growth control and glucose metabolism of cancer cells. The PI3Ks include three major classes of lipid kinases, termed class I (including Ia and Ib), class II, class III, and a distantly related Class IV [118]. Akt (also known as protein kinase B) is a serine/threonine protein kinase directly activated by PI3K [119]. The PI3K/Akt pathway regulates a broad range of normal cellular processes. However, many studies have shown that this pathway is altered in cancer cells and promotes the survival, proliferation, growth, metabolism, angiogenesis and metastasis of cancer [120]. For example, in HCC, the PI3K/Akt pathway activation promotes angiogenesis and EMT via exosomes and microRNA-32-5p [121]. The PI3K/Akt pathway activation also accounts for sorafenib resistance and induces multidrug resistance in HCC [122]. Using LY294002, a PI3K inhibitor, sorafenib resistance can be reversed in HCC [123].

The PI3K/Akt signaling pathway can regulate the glycolysis in HCC through the following mechanisms. Firstly, activation of the PI3K/Akt signaling pathway can induce the expression of GLUT1 and GLUT4 to increase the glucose uptake rate in cancer cells (Fig. 3) [124, 125]. It has been reported that the PI3K/Akt pathway can also promote the translocation of GLUT1 and GLLUT4 from the cytoplasm to the cell membrane [126, 127]. Secondly, the PI3K/Akt pathway can regulate the activity or the expression of some glycolytic enzymes, such as HK2, PFK1 and PFK2. For example, activation of PI3K/Akt pathway can promote the binding of HK2 to VDAC in mitochondria and increase HK2 activity directly [128]. PI3K/Akt activation can also directly cause the phosphorylation and activation of PFK2, leading to increased production of F-2, 6-BP to further enhance PFK1 activity [129]. Thirdly, PI3K/Akt can indirectly regulate the expression of glycolytic enzymes by interacting and regulating the expression of AMPK and HIF-1α [14, 35]. Fourthly, PI3K/Akt also activates the downstream regulator of the mammalian target of rapamycin (mTOR) to further activate HIF-1α, so as to promote aerobic glycolysis, angiogenesis and neo-vascularization in cancer cells [18].

### HIF-1α

As mentioned above, because of the rapid proliferation and expansion of cancer cells, hypoxia is present in the core of tumor tissues. In 1992, Semenza et al. first reported the nuclear transcription factor induced by hypoxia in Hep3B HCC cell line, which was termed as HIF-1α and acted as an enhancer of erythropoietin (EPO) gene [130]. HIF-1α can be stabilized by hypoxia and then can bind to the hypoxia responsive elements (HRE) of target genes’ promoters, resulting in the transcription of related genes involved in overcoming hypoxic effects [49]. HIF-1α plays a key role in the regulation of proliferation, glucose metabolism, angiogenesis, invasion and metastasis, and multidrug resistance in cancer cells [9]. HIF-1α is commonly overexpressed in HCC patients, and the higher expression levels of HIF-1α are correlated to poor prognosis. It has been reported that HIF-1α can control the transcriptional expression of over 80 genes that are involved in glucose metabolism, cell survival, angiogenesis, invasion and metastasis [131, 132]. By inhibiting or interfering with the expression of HIF-1α, it is effective to inhibit the energy metabolism and growth of HCC.

As a key regulator of glycolytic metabolism in HCC, HIF-1α activation also contributes to the regulation of the Warburg effect, mostly at the transcriptional level as follows. Firstly, as a transcription factor, HIF-1α can promote the transcription of GLUT-1, thereby enhancing the glucose uptake for glycolysis [133]. Secondly, HIF-1α can control the transcription of several glycolytic enzymes, including HK2, PFK1, PKM2, LDHA and glyceraldehyde-3-phosphate dehydrogenase (GAPDH). By upregulating the expression of these genes, the glycolysis level is further enhanced [134]. Thirdly, HIF-1α can promote the expression of pyruvate dehydrogenase kinase (PDK) to inhibits PDH activity, leading to the conversion of pyruvate into lactate [135]. Moreover, except for hypoxic stress, HIF-1α can also be regulated by various signaling pathways, including PI3K/Akt/mTOR, Raf/MAPK and AMPK, causing glycolysis levels to accelerate (Fig. 3) [136].

### C-Myc

c-Myc is the most common oncogene involved in human carcinogenesis, and takes part in the regulation of cell cycle, cellular survival, proliferation and metabolic reprogramming [137]. Genetic analyses have demonstrated that c-Myc over-expression is seen in up to 70% of viral and alcohol-related HCC [138]. Moreover, c-Myc overexpression has been reported to cause liver tumors generation in mice with enhanced glycolysis levels [139]. Like HIF-1α, c-Myc is another vital oncogene involved in the Warburg effect in HCC.

The oncogene c-Myc is known to positively regulate the Warburg effect through the following mechanisms. Firstly, as a key transcription factor, c-Myc was first linked to glycolysis because it can transactivate and promote the expression of LDH just like HIF-1α [140]. Secondly, c-Myc can also promote the expression of GLUT1, PKM2 and HK2 to promote the glycolysis flux [135, 141]. When bound to the PKM2 promoter, c-Myc can not only induce PKM2 expression, but also promote the PKM2/PKM1 ratio to promote the survival of cancer cells. Besides, the nuclear PKM2 can also act as a coactivator of β-catenin to induce c-Myc expression in turn, resulting in a positive feedback loop to promote the sustained expression of glycolytic genes sustaining [142]. Thirdly, c-Myc can activate PDK1 in collaboration with HIF-1α, causing increased lactate production and the acidity of the extracellular environment [143]. Fourthly, c-Myc has been suggested to mediate the elevation of glutaminolysis in cancer cells by promoting both the glutamine uptake and glutamine catabolism, thereby maintaining the integrity in mitochondrial TCA cycle to promote the survival of cancer cells [144]. In addition, there is also close interaction between c-Myc and other signaling pathways and transcription factors, such as HIF-1α, β-catenin, JAK/STAT3 signaling pathway and ERK signaling pathway (Fig. 3) [141, 143, 144].

Based on the critical role of c-Myc in HCC carcinogenesis, it is obvious that c-Myc is another attractive target for the development of a novel therapy. Lin et al. used a small molecule inhibitor of c-Myc, which was 10,058-F4, to treat HCC cell lines, and the results showed that this molecule could inhibit the proliferation and enhance chemosensitivity of HCC cells to low-dose doxorubicin, 5-fluorouracil and cisplatin [145]. There is another small molecule compound CX3543 (also called Quarfloxin), which targets and inhibits Myc G-quadruplexes, is currently in phase II clinical trials for the treatment of neuroendocrine carcinomas [146].

### Noncoding RNAs

Noncoding RNAs are groups of functional RNAs that cannot be transcribed into proteins, but instead take part in various biological processes to regulate gene transcription and translation. Noncoding RNAs are responsible for up to 98% of the whole genome’s transcripts, and they includes microRNAs (miRNAs), long non-coding RNAs (Lnc-RNAs), small interfering RNAs (siRNAs) and small nuclear RNAs (snRNAs) [147]. Noncoding RNAs, especially miRNAs and Lnc-RNAs, have been found to regulate the Warburg effect, and mainly through the regulation of glycolytic enzymes expression or glycolysis related pathways [35].

The three rate-limiting enzymes in the glycolysis pathway can all be regulated by various noncoding RNAs (Table 1). The miR-34a for example is downregulated in metastatic HCC tissues, and has been reported to inhibit HCC glycolysis by directly targeting HK1, HK2, and glucose-6-phosphate isomerase (GPI) [153]. The miR-199a-5p is also down-regulated in HCC tissues, and is associated with the tumor size and prognosis of patient. The miR-199a-5p can be inhibited by HIF-1α, leading to the inhibition of HK2 expression and the disruption of liver metabolism [157]. The miR-139-5p was found to regulate HK1 and PFKFB3 expression to regulate glycolysis, proliferation, migration and invasion of HCC [152]. Moreover, by directly targeting HK2, miR-125b was found to downregulate glucose metabolism so as to relieve 5-FU resistance in HCC cells [154]. Some Lnc-RNAs, such as Lnc-TUG1, have also been demonstrated to regulate HK2 expression through the TUG1/miR-455-3p/AMPKb2 axis [156]. Furthermore, miR-520 can inhibit the expression of PFK1 through Tat-activating regulatory DNA-binding protein (TARDBP)-mediated regulation of glycolysis in HCC [160]. PKM2 can also be regulated by miRNAs and Lnc-RNAs, for example, miR-122 is down-regulated in HCC tissues, therefore, overexpression of miR-122 in HCC cells can inhibit the expression of PKM2 to promote apoptosis and inhibit migration and invasion of Hep3B cells [161]. The miR-491–5p also functions as a tumor suppressor in HCC by reducing PKM2 expression [164]. The miR-374b is also involved in reducing the expression of PKM2 by inhibiting the expression of hnRNPA1, which causes re-sensitization of sorafenib HCC cells [165]. The Lnc-RNA LINC01554 is often downregulated in HCC, and it’s revealed that LINC01554 promotes the degradation of PKM2 through Akt/mTOR signaling pathway [163]. Moreover, a circular RNA, circMAT2B can also increase the expression levels of PKM2 through the regulation of the miR-338-3p [162].

**Table 1:** Noncoding RNAs and their targets in aerobic glycolysis in HCC

Target	ncRNA	In vivo or in vitro	Involvement of other factors	Reference
GLUT	miR-342-3p	both	PI3K/AKT	[148]
	miR-505	both	IGF-1R	[149]
	miR-455-5p	both	IGF-1R/AKT	[150]
	lncRNA HOTAIR	both	mTOR	[151]
HK2、HK1	miR-139-5p	both	ETS1	[152]
	miR-34a	in vitro	p53	[153]
	miR-125b	both	–	[154, 155]
	Lnc-TUG1	in vitro	TUG1/miR-455-3p/AMPKβ2	[156]
	miR-199a-5p	both	PKM2	[157, 158]
	miR-885-5p	both	HIF-1α	[159]
PFK1、PFKFB3	miR-139-5p	both	ETS1	[152]
	miR-520	both	TARDBP	[160]
PKM2	miR-122	both	–	[161]
	circMAT2B	both	miR-338-3p	[162]
	lncRNA LINC01554	both	Akt/mTOR	[163]
	miR-491–5p	both	–	[164]
	MiR-374b	in vitro	hnRNPA1	[165]
LDHA	miR-142-3p	both	–	[166]
	miR-34a	both	–	[167, 168]
HIF-1A	miR-199a-5p	both	–	[169]
	miR-3662	both	ERK/JNK	[170]
PI3K/Akt/mTOR	miR-125a	in vitro	–	[171]
	miR-7	both	–	[172]
c-myc/STAT3	miR-23a	in vitro	PEPCK	[173]
	miR-129-5p	both	PDK4	[174]
JAK/STAT	miR-196a /b	both	SOCS2	[175]
PPARγ	lncRNA Ftx	both	–	[176]
mTOR/TCF7L2	lncRNA MALAT1	in vitro	mTOR	[177]

Other key proteins, such as GLUT1 and LDHA are also regulated by noncoding RNAs in HCC, leading to changes of glucose uptake and lactate production levels. For example, miR-342-3p, miR-455-5p and miR-505 are all down-regulated in HCC tissues, and they have been reported to be able to decrease glycolysis by inhibiting GLUT1 expression through insulin growth factor receptor 1 (IGF-1R) or the PI3K/AKT pathway [148–150]. Whereas Lnc- HOTAIR was found to promote glycolysis by upregulating GLUT1 expression through mTOR mediated pathway [151]. Furthermore, miR-142-3p and miR-34a have been found to target LDHA to suppress aerobic glycolysis and cell proliferation in HCC [166–168].

Noncoding RNAs can also regulate glycolysis levels in HCC by targeting various signaling pathways involved in glycolysis. For example, HIF-1α, the key regulator in response to hypoxia, can be directly regulated by miR-199ab-5p and miR-3662 to inhibit the Warburg effect in HCC [169, 170]. While the miR-125a and miR-7 can suppress HCC progression by inhibiting the PI3K/Akt pathway [171, 172]. The c-Myc is the target of miR-129-5p and miR-23a [174, 178], and mTOR is the target of lncRNA MALAT1 [177], and PPAR-γ is the target of lncRNA Ftx [176]. Through the interaction with the STAT3 pathway, miR-196a or miR-196b is able to suppress cell proliferation and glycolysis, and induced apoptosis in HCC cells [175].

## Discussions and expectations

The metabolic shift from OXPHOS to aerobic glycolysis is not only a hallmark of HCC, but also provides many potential targets for exploitation in in HCC therapy. After an appreciation of the important roles of aerobic glycolysis in HCC, an understanding of the regulatory mechanisms involved will enable researchers the opportunity to develop novel therapeutic methods. These may include: (1) targeting the three rate-limiting enzymes in glycolysis to inhibit the aerobic glycolysis process directly. (2) Targeting the GLUTs or LDHA to inhibit the glucose uptake or lactate levels. (3) Targeting the regulatory factors and signaling pathways involved in glycolysis to regulate glycolysis indirectly. Moreover, sorafenib as a target drug for HCC, was found to induce drug resistance which includes the participance of enhanced glycolysis. If combined with glycolysis targeting drugs, such as 2-DG, the anti-cancer effect of sorafenib may be enhanced, and sorafenib resistance can also be reversed [91].

### Therapeutic agents that target glycolysis

Currently, there are some widely-used agents that can target on HK2, PFK1 or PKM2 to inhibit glycolysis. For example, 2-DG is an analog of glucose, and it can be catalyzed by HK2 into 2-deoxy-D-glucose-6-phosphate (2-DG-6P), which is different from G-6-P and can noncompetitively inhibit the activity of HK2 [5, 35]. When 2-DG was given alone in HCC, it was reported to suppress the growth, metastasis and invasion of HCC cells, and the combination treatment of 2-DG and other chemotherapy drugs, such as sorafenib, 2-aminophenoxazine-3-one (Phx-3), and metformin, can also enhance the anticancer effect of them on HCC [179–181]. The 3-bromopyruvate (3-BP) is a different type of HK2 inhibitor with the ability to inhibit HK2 activity directly, so as to strongly suppress the glycolysis process. Many in vitro and in vivo studies have verified the anti-HCC effects of 3-BP, and recently it has been approved by the FDA to treat HCC and intrahepatic cholangiocarcinoma [182]. The 3-(3-pyridinyl)-1-(4-pyridinyl)-2-propen-1-one (3-PO) is a selective PFKFB3 inhibitor and has been found to inhibit the growth and glycolysis of lung cancer and breast cancer both in vivo and in vitro [183]. Moreover, there are also some effective inhibitors of PKM2, such as TT-232(CAP-232) and shikonin, that can inhibit the growth and induce apoptosis of cancer cells [184–186].

However, although some of these drugs targeting glycolysis are currently undergoing phase I or II clinical trials, their clinical application is far from secure. Besides, the design of metabolic targeted therapeutic strategies should also evaluate the tumor heterogeneity and interaction with the micro-environment carefully, giving rise to the difficulties in development of effective drugs. Therefore, the exploration of new agents, especially traditional Chinese medicine monomers, such as genistein, 15d-PGJ2, quercetin, and oleanolic acid, to test for anti-glycolysis effects are urgently needed with the help of proteomics and metabolomics analyses and genome-scale metabolic models [26, 187–190].

### Anti-VEGFR or anti-PD-1/PD-L1 agents

As mentioned above, the inducing of sorafenib resistance in HCC treatment is correlated with the enhanced aerobic glycolysis levels, which limits the application and availability of sorafenib. Therefore, recently, there are some other agents, which are also receptor tyrosine kinase (RTK) inhibitors as sorafenib that can inhibit VEGFR activities and angiogenesis of HCC, having been used in several phase III trials as first-line or second-line chemotherapy for HCC to determine whether these agents are superior to sorafenib. For example, lenvatinib has been tested as first line treatment in the REFLECT trial, and was shown to be non-inferior to sorafenib (overall survival) [191]. While regorafenib, cabozantinib, and ramucirumab were shown to be superior to placebo in HCC patients failing sorafenib treatment [192, 193]. A phase II trial that combined bevacizumab (VEGFR inhibitor) and erlotinib (EGFR inhibitor) was also designed to compare the effect as first-line treatment for advanced HCC patients to sorafenib alone, and the results showed that there was no difference in efficacy between the bevacizumab + erlotinib group and sorafenib alone, while bevacizumab + erlotinib showed a better safety and tolerability [194]. Therefore, these anti-VEGFR agents are considered as “preferred regimens” as first line treatment for advanced HCC [195].

The programmed death-1 (PD-1) is a famous immune checkpoint, which can be activated by its ligands, such as PD-L1, leading to the suppression of immune response. During carcinogenesis, cancer cells take advantage of the PD-L1/PD-1 system to evade immune supervision. It has been reported that compared with PD-L1-negative HCC patients, PD-L1-positive HCC patients showed significantly worse overall survival [196]. Blocking PD-1 or PD-L1 has become a therapeutic method for HCC treatment, such as atezolizumab, pembrolizumab and nivolumab. Most recently, Finn and his colleagues conducted a phase III IMbrave150 trial, which compared the combination of bevacizumab and atezolizumab (targeting programmed cell death ligand 1, PD-L1) against standard sorafenib treatment in first line treatment for advanced HCC. The current results showed that the combination can increase the progression-free survival and overall survival than patients who received sorafenib alone [197–199]. Therefore, the NCCN guideline (2020) also included the combination of bevacizumab and atezolizumab as another first-line standard treatment for unresectable HCC just as sorafenib.

### Role of androgen receptors and induction of glycolysis in HCC

HCC is a considered as a male-dominant cancer. Zhang et al. reported in 2018 that androgen receptor (AR) was overexpressed in the nucleus of 37% of HCC tumors, which may be correlated to the advanced disease stage and poor survival of HCC patients [200]. The role of AR expression in HCC has been investigated by many researchers. Wang et al. found that AR can promote HBV viral RNA expression, which favors HBV-driven hepatocarcinogenesis [201]. Ma et al. found that AR knockout reduced liver tumors number and burden in mice, suggesting that AR is necessary for full cancer development. The activation of AR may lead to the activation of androgen response elements, and then the transcription of target genes, including Src, EGFR, ERK, HIF-1α, and CREB [202].

AR has also been found to induce glycolysis in HCC. Sun et al. found that AR expression is positively correlated with HK2 levels, and then promoted HCC growth by enhancing HK2-mediated glycolysis through the regulation of PKA/CREB [203]. Moreover, Zhang et al. demonstrated that AR can crosstalk with mTOR, which may also help the activation of glycolysis through mTOR mediated pathway [200].

Antiandrogen and anti-AR agents, such as bicalutamide and enzalutamide, are therefore considered as therapeutic methods for HCC treatment as well. Unfortunately, early clinical trials on anti-androgen and bicalutamide therapies in liver cancer met with disappointing results, producing no apparent clinical benefits [204, 205]. But, the combination of anti-AR and other chemotherapy agents, such as sorafenib, may be a new approach for HCC treatment. For example, Xiao et al. reported that found that AR can re-sensitize HCC to sorafenib through AR/miR-520f-3p/SOX9 signaling [206]. Jiang et al. observed that sorafenib-induced apoptosis can be enhanced by AR inhibition in HCC cell lines [207]. Wang et al. also reported that sorafenib inhibited AR activation induced by HBx [208]. Taken together, based on the role of AR on HCC growth and glycolysis, AR is still a promising target for HCC treatment.

### Effects of fasting on glycolysis regulating genes and apoptosis

Recently years, fasting/starvation (16–60 h) has also been tested in some clinical trials for cancer treatment because it has been found that fasting can reduce the side effects associated to high dose chemotherapy in various cancer patients, and protect normal cells from injuries (NCT01304251, NCT01175837, NCT00936364, NCT01175837) [209]. However, the molecular mechanisms how fasting suppresses cancer cells remain unknown.

Liver is the primary organ for glucose metabolism. During fasting, approximately 80% of endogenous glucose is produced through gluconeogenesis, which is actually a reverse pathway of glycolysis, to adapt to stress conditions [210]. Valentina Sukonina et al. found that 16 h of fasting could induce the elevation of glycolysis in mice, with enhanced glucose uptake levels and impaired OXPHOS, mainly through FOXK1 and FOXK2 mediated glycolytic gene expression, including HK2, PFKM, PKM2 and LDHA [211]. However, in cancer cells, which are highly depended on glucose for energy supply, the situation is totally opposite. It has been reported that deprivation of glucose (fasting) impairs glycolysis and the pentose phosphate pathway, induces oxidative stress because of enhanced production of reactive oxygen species (ROS), resulting in the redox imbalance and then cancer cell death [106]. Moreover, Bianchi et al. reported that fasting could induce anti-Warburg effect in colon cancer models, including the downregulation of HK2, PFK, PKM2, and LDH expression. Moreover, they also found that because of the increase in mitochondrial respiration, fasting can also promote apoptosis in colon cancer cell [212]. Grasl-Kraupp have also reported in 1994 that fasting could eliminate preneoplastic cells through apoptosis, thereby inhibiting the carcinogenesis in rat liver [213]. The mechanisms how fasting induces apoptosis in HCC cells may include: (1) fasting improves OXPHOS in mitochondria, which then enhances the electron transport chain function and the production of ROS, leading to the induction of apoptosis [212]. (2) Fasting induces inducible nitric oxide synthase and interferon-γ production, so as to increase ROS production and induce apoptosis [214].

In conclusion, owing to the functional role of fasting on HCC glycolysis and apoptosis, fasting is also a potential therapeutic approach for HCC treatment, and we can pay more attention to it.

### Role of EMT in HCC

As mentioned above, EMT is responsible for sorafenib resistance, as well as HCC angiogenesis and metastasis. EMT of cancer cells indicates that epithelial cells lose their cell-cell adhesions and apicobasal polarity, and acquire more mesenchymal and invasive/metastatic phenotype [215]. EMT also plays important role in the aerobic process in HCC. Zhang et al. found that the depletion of mitochondrial fusion protein mitofusin-1 (MFN1) triggered the EMT of HCC and modulated HCC metastasis by metabolic shift from aerobic glycolysis to oxidative phosphorylation [216]. EMT also contributes to the increased population of cancer stem-like cells (CSCs) associated with Wnt/β-catenin activation, and can lead to tumor heterogeneity and therapeutic resistance [217]. In liver tissues, hepatocytes generally harbor the rapid cellular proliferation under both normal and inflammatory conditions. It still remains unknown whether the origin of HCC is from the CSCs or differentiated hepatocytes [215].

The hepatic stellate cells (HSCs) are kinds of mesenchymal cells in liver tissues, and can transdifferentiate into myofibroblasts in response to stimulus. Duran et al. reported that p62/SQSTM1, which can interact with the components of the mTORC1 complex during autophagy process, can negatively control HSCs activation through binding to the vitamin D receptor (VDR) [218]. Through acting as carcinoma-associated fibroblasts (CAFs), HSCs undergo EMT both in liver fibrosis, cirrhosis and HCC, leading to the tumor formation and development [219]. Taken together, the EMT process and the myofibroblasts in liver tissues are another therapeutic target for HCC treatment.

## Conclusions

In conclusion, the aerobic glycolysis plays important roles in the progression of HCC, including proliferation, immune evasion, invasion and metastasis, angiogenesis, and drug resistance. Via targeting key factors included in aerobic glycolysis, such as the inhibition of enzymes HK2, PFK or PKM2, and the regulatory pathways. Represent potential new therapeutic approaches for the treatment of HCC.

## Data Availability

Not applicable.
